# Frailty Index and Risk of Ischemic Stroke in China: Evidence from a Cohort Study, Disease Burden Analysis, and Mendelian Randomization

**DOI:** 10.3390/healthcare14131932

**Published:** 2026-07-01

**Authors:** Yanlong Zhou, Dongdong Jia, Zengcai Liu, Yinju Liu, Lanying Chen

**Affiliations:** 1 National Pharmaceutical Engineering Center for Solid Preparation of Chinese Herbal Medicine, Jiangxi University of Chinese Medicine, Nanchang 330006, China; zhouyanlong@jxutcm.edu.cn (Y.Z.); jiadongdong@jxutcm.edu.cn (D.J.); liucengcai@jxutcm.edu.cn (Z.L.); liuyinju@jxutcm.edu.cn (Y.L.); 2 International Education College, Jiangxi University of Chinese Medicine, Nanchang 330006, China; 3 Jiangxi Provincial Key Laboratory of Effective Material Basis of TCM, Jiangxi University of Chinese Medicine, Nanchang 330004, China

**Keywords:** frailty index, ischemic stroke, cohort study, China, Mendelian randomization

## Abstract

**Objective:** This study aims to examine the association between the frailty index (FI) and stroke risk among Chinese adults, describe the burden of stroke in China, and explore the causal role of FI in ischemic stroke through Mendelian randomization. **Methods:** Data from the China Health and Retirement Longitudinal Study (CHARLS) included 13,473 participants aged 45 years and older without a history of stroke. Cox models, restricted cubic splines, and sensitivity analyses were employed to assess the association between the modified frailty index (mFI) and incident stroke. Additionally, data from the Global Burden of Disease (GBD) 2021 data report were utilized to describe stroke trends in China from 1990 to 2021. Two-sample Mendelian randomization was conducted to evaluate the causal effects of FI on ischemic stroke subtypes. **Results:** During a median follow-up period of approximately 7 years, 811 incident strokes were recorded. Each 0.1-point increase in mFI was associated with a 16% increase in stroke risk (HR = 1.16, 95% CI: 1.06–1.27), demonstrating a linear dose–response relationship. From 1990 to 2021, the proportion of ischemic stroke rose from 46.9% to 63.2%. Mendelian randomization analysis provided genetic evidence supporting a causal association between FI and ischemic stroke (OR = 1.191, 95% CI: 1.046–1.357), particularly driven by large-artery atherosclerotic (OR = 1.852) and small-vessel stroke (OR = 1.415), but not by cardioembolic stroke. **Conclusions:** A higher FI is associated with an increased risk of stroke among Chinese adults, with genetic evidence supporting a causal role in ischemic stroke. Therefore, FI may serve as a valuable addition to existing risk assessment tools.

## 1. Introduction

Stroke remains a major public health challenge, and its burden continues to increase with rapid population aging [1]. This trend is especially pronounced in China, where a large population, accelerated aging, and a high prevalence of metabolic disorders and cardiovascular risk factors collectively contributed to the rising incidence of stroke [2,3]. In this context, identifying early high-risk states and upstream determinants is essential for enhancing primary prevention.

Frailty is an age-related clinical syndrome characterized by a decline in physiological reserve across multiple systems, resulting in reduced resilience to internal and external stressors, which renders individuals more vulnerable [4,5]. Unlike single diseases or isolated risk factors, frailty reflects the cumulative effects of biological aging and overall health deterioration. Previous studies have demonstrated that frailty is closely associated with a range of adverse outcomes, including falls [6], hospitalization [7], disability, cardiovascular events, and mortality [8]. To quantify this complex condition, several assessment approaches have been developed. Among these, the frailty index (FI), based on the deficit accumulation model, captures the extent of health deficits as a continuous measure and has been widely utilized in population-based studies [9,10].

In recent years, FI has increasingly been utilized to assess overall health status, generating growing interest in its predictive value for adverse cardiovascular outcomes. Emerging evidence indicates that a higher FI may be associated with an elevated risk of stroke [11]. From a biological standpoint, the accumulation of health deficits captured by the FI is frequently accompanied by chronic low-grade inflammation, vascular aging, metabolic dysregulation, and neuroendocrine imbalance, all of which may contribute to the development of cerebrovascular events [12,13]. However, the current body of evidence is predominantly derived from observational studies, which are subject to significant limitations [14,15,16]. Cross-sectional and retrospective designs restrict the ability to establish temporal relationships, and even prospective studies cannot entirely eliminate residual confounding from comorbidities and lifestyle factors. Furthermore, subclinical vascular changes that precede a stroke may already influence FI levels, raising the possibility of reverse causation. Consequently, it remains uncertain whether the FI serves as an independent risk factor for stroke or merely a concomitant manifestation of the disease process. Additionally, systematic reviews are warranted to ascertain whether the FI can reliably predict stroke risk, particularly ischemic stroke and its subtypes, and whether this association is substantiated by genetic evidence.

Revisiting this question within the Chinese population is particularly relevant [17]. Over the past few decades, the burden of stroke in China has steadily increased, accompanied by shifts in subtype distribution, with ischemic stroke accounting for an ever-growing proportion [18]. In this context, evaluating the association between FI and stroke risk by integrating individual-level prospective evidence with national-level burden data may yield a more comprehensive understanding of its public health implications. As the burden of ischemic stroke continues to escalate, identifying upstream risk factors may provide additional insights for enhancing prevention strategies.

While conventional observational studies can characterize the association between FI and stroke, their capacity to support causal inference is limited. Mendelian randomization, which utilizes genetic variants associated with the exposure as instrumental variables, presents an approach that is less vulnerable to residual confounding and reverse causation [19]. For a complex phenotype such as the frailty index, Mendelian randomization offers an opportunity to examine whether a genetic predisposition to a higher FI is linked to ischemic stroke and its subtypes.

In this context, the cohort analysis was conducted to evaluate the prospective association between FI and stroke risk at the individual level in a longitudinal population-based setting. The Global Burden of Disease (GBD) analysis was included to provide a broader epidemiological perspective by describing temporal trends and subtype distribution of stroke in China, thereby contextualizing the cohort findings and highlighting the population-level relevance of the study question. Finally, Mendelian randomization was performed to provide complementary genetic evidence regarding potential causality, helping to reduce bias arising from confounding and reverse causation.

## 2. Methods

### 2.1. Study Design

We conducted a multi-level study that integrated observational, population-based, and genetic analyses. A prospective cohort from the China Health and Retirement Longitudinal Study (CHARLS) was utilized to investigate the association between the frailty index (FI) and the incidence of stroke in individuals aged 45 years and older. Data from the Global Burden of Disease (GBD) 2021 study were employed to describe temporal trends in stroke incidence, age distribution, and subtype composition in China from 1990 to 2021. Additionally, a Mendelian randomization analysis was conducted to examine the potential causal effect of the FI on ischemic stroke.

### 2.2. Epidemiological Analysis: CHARLS Cohort

#### 2.2.1. Study Population and Exclusion Criteria

**Figure 1:**
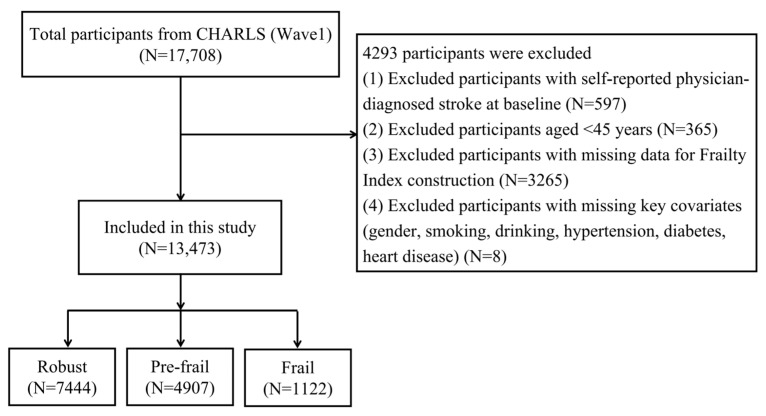
Flow diagram of participant selection from the China Health and Retirement Longitudinal Study (CHARLS).

Data were obtained from the China Health and Retirement Longitudinal Study (CHARLS) [20], a nationally representative prospective cohort of Chinese adults aged 45 years and older. The study commenced with a baseline in 2011, including 17,708 participants who were followed from 2013 to 2018 to identify incident stroke events. The exclusion criteria were as follows: (1) self-reported physician-diagnosed stroke at baseline; (2) age under 45 years; (3) insufficient data to construct the FI; and (4) missing key covariates. Consequently, a total of 13,473 participants were included in the final analysis (Figure 1). Baseline characteristics were compared between included and excluded participants to assess potential selection bias. Stroke events were identified through follow-up surveys, with time-to-event defined from baseline to the first report; participants without stroke were censored at their last follow-up.

#### 2.2.2. Construction of the FI and Modified Frailty Index (mFI)

The FI was constructed based on the deficit accumulation model, utilizing 38 health deficits encompassing chronic conditions, symptoms, functional limitations, and objective measurements [21]. Each item was coded either 0 or 1, and the FI was calculated as the proportion of deficits present, ranging from 0 to 1, with higher values indicating greater frailty [22]. To ensure reliability, the FI was calculated only for participants with at least 70% of items available. Participants were categorized as robust (FI ≤ 0.10), pre-frail (0.10 < FI < 0.25), or frail (FI ≥ 0.25) (Supplementary Table S1).

To mitigate potential overadjustment arising from the inclusion of overlapping cardiovascular-related conditions as both components of the FI and covariates in the fully adjusted models, a modified FI (mFI) was constructed by excluding hypertension, diabetes, dyslipidemia, and heart disease from the index. The mFI was then recalculated using the same deficit accumulation approach and analyzed as a continuous variable.

FI categories were employed for descriptive and Kaplan–Meier analyses, while the mFI served as the primary exposure in regression, spline, and sensitivity analyses. Sensitivity analyses using the mFI yielded materially similar results to those of the primary analyses, indicating that the study findings were robust to the modified frailty index.

#### 2.2.3. Outcome Definition and Statistical Analysis

The primary outcome was defined as incident stroke, characterized by the first self-reported physician diagnosis during the follow-up period. Continuous variables are expressed as mean ± standard deviation or median (interquartile range), whereas categorical variables are presented as counts (percentages). Differences between groups were evaluated using ANOVA or Kruskal–Wallis tests, along with χ^2^ tests.

Cox proportional hazards models were utilized to estimate hazard ratios (HRs) and 95% confidence intervals (CIs) [23], with sequential adjustments for demographic, lifestyle, and clinical factors. Model 1 was unadjusted; Model 2 included adjustments for age and sex; Model 3 further incorporated adjustments for education, marital status, smoking status, alcohol consumption, body mass index, depressive symptoms, hypertension, diabetes, dyslipidemia, and cardiovascular disease.

Kaplan–Meier curves were employed to compared stroke-free survival across frailty categories [24], and restricted cubic splines (with four knots) were used to examined dose–response relationships. Sensitivity analyses excluded early events occurring within the first two years and baseline heart disease. The proportional hazards assumptions were verified using Schoenfeld residuals. A two-sided *p*-value of less than 0.05 was deemed statistically significant.

### 2.3. GBD Data Analysis

#### 2.3.1. Data Source and Study Scope

Stroke burden data were sourced from the Global Burden of Disease (GBD) 2021 study, published by the Institute for Health Metrics and Evaluation (IHME). Data pertaining to China from 1990 to 2021 were extracted using the GBD Results Tool (https://vizhub.healthdata.org/gbd-results/) (accessed on 29 June 2026) [25]. The analysis encompassed overall stroke as well as its three principal subtypes: ischemic stroke, intracerebral hemorrhage, and subarachnoid hemorrhage.

#### 2.3.2. Measures and Statistical Analysis

The number of incident cases and the crude incidence rate (per 100,000 population) were utilized to describe temporal trends in stroke burden within China. Trends in overall stroke incidence from 1990 to 2021 were initially assessed, followed by an examination of the age-specific distribution of incident cases in 2021. Subsequently, changes in the composition of stroke subtypes over time were analyzed to characterize shifts in the stroke spectrum.

### 2.4. Genetic Causal Inference: Mendelian Randomization

#### 2.4.1. Data Sources and Instrument Selection

Genetic instruments for the FI were derived from a genome-wide association study (GWAS) involving 175,226 individuals of European ancestry [26]. Summary statistics for ischemic stroke and its subtypes—namely, small-vessel stroke, large-artery atherosclerotic stroke, and cardioembolic stroke—were obtained from published GWAS datasets [27]. Single nucleotide polymorphisms (SNPs) were selected based on genome-wide significance (*p* < 5 × 10^−8^), with a more relaxed threshold (*p* < 5 × 10^−6^) applied when necessary [28], and were clumped for independence (r^2^ < 0.001, 10,000 kb). Palindromic SNPs were excluded, and the strength of the instruments was evaluated using the F-statistic (F > 10). Reverse Mendelian randomization was conducted to investigate potential reverse causation, while multivariable Mendelian randomization further adjusted for systolic blood pressure and body mass index (Supplementary Table S2).

#### 2.4.2. Two-Sample Mendelian Randomization Analysis

A two-sample Mendelian randomization framework was employed, utilizing inverse-variance weighted (IVW) analysis as the primary method. Complementary analyses, including MR-Egger, weighted median, weighted mode, and simple mode, were performed evaluate robustness and pleiotropy [29,30,31,32].

#### 2.4.3. Sensitivity Analyses and Heterogeneity Assessment

Heterogeneity was assessed using Cochran’s Q statistic, with random-effects IVW applied when heterogeneity was present. Directional pleiotropy was evaluated using the MR-Egger intercept and MR-PRESSO. Outliers identified by MR-PRESSO were excluded if distortion was detected, followed by re-estimation [33]. A leave-one-out analysis was conducted to evaluate the influence of individual SNPs [34]. The primary IVW results were corrected for multiple testing using a false discovery rate (FDR) threshold of <0.05, whereas sensitivity analyses were not adjusted. All Mendelian randomization analyses were performed using R software (version 4.5.1; R Foundation for Statistical Computing, Vienna, Austria).

## 3. Results

### 3.1. Association Between Frailty Index and Stroke Risk: Evidence from the CHARLS Cohort

#### 3.1.1. Baseline Characteristics

A total of 13,473 participants were included in this study and categorized into three groups based on their baseline frailty index (FI): robust, pre-frail, and frail. Compared to the robust group, participants in the frail group were older, more likely to be female, had lower educational attainment, and exhibited a higher prevalence of comorbidities, including hypertension, diabetes, dyslipidemia, and cardiovascular disease (all *p* < 0.001; Table 1). During a median follow-up period of approximately 7 years, 811 incident stroke events were recorded. The baseline characteristics of both included and excluded participants are presented in Supplementary Table S3 to assess potential selection bias.

#### 3.1.2. Association Between Frailty Index and Stroke Risk

Cox proportional hazards models indicated that a higher FI was associated with an increased risk of stroke (Table 2). In the unadjusted model, each 0.1 increase in FI was associated with a 44% higher risk of stroke (HR = 1.44, 95% CI: 1.35–1.54; *p* < 0.001). This association remained significant after adjusting for age and sex (HR = 1.41, 95% CI: 1.31–1.51; *p* < 0.001). In the fully adjusted model, which utilized the modified frailty index (mFI), each 0.1 increase in mFI was associated with a 16% higher risk of stroke (HR = 1.16, 95% CI: 1.06–1.27; *p* = 0.0015).

Sensitivity analyses yielded consistent results. After excluding participants who experienced stroke events within the first 2 years of follow-up, the association remained significant (HR = 1.18, 95% CI: 1.07–1.30; *p* = 0.001). Further exclusion of participants with baseline heart disease did not materially alter the results (HR = 1.22, 95% CI: 1.10–1.35; *p* < 0.001). No violation of the proportional hazards assumption was detected for mFI (Schoenfeld test *p* = 0.305), and the global test similarly indicated no significant deviation (*p* = 0.208).

#### 3.1.3. Kaplan–Meier Analysis and Dose–Response Relationship

**Figure 2:**
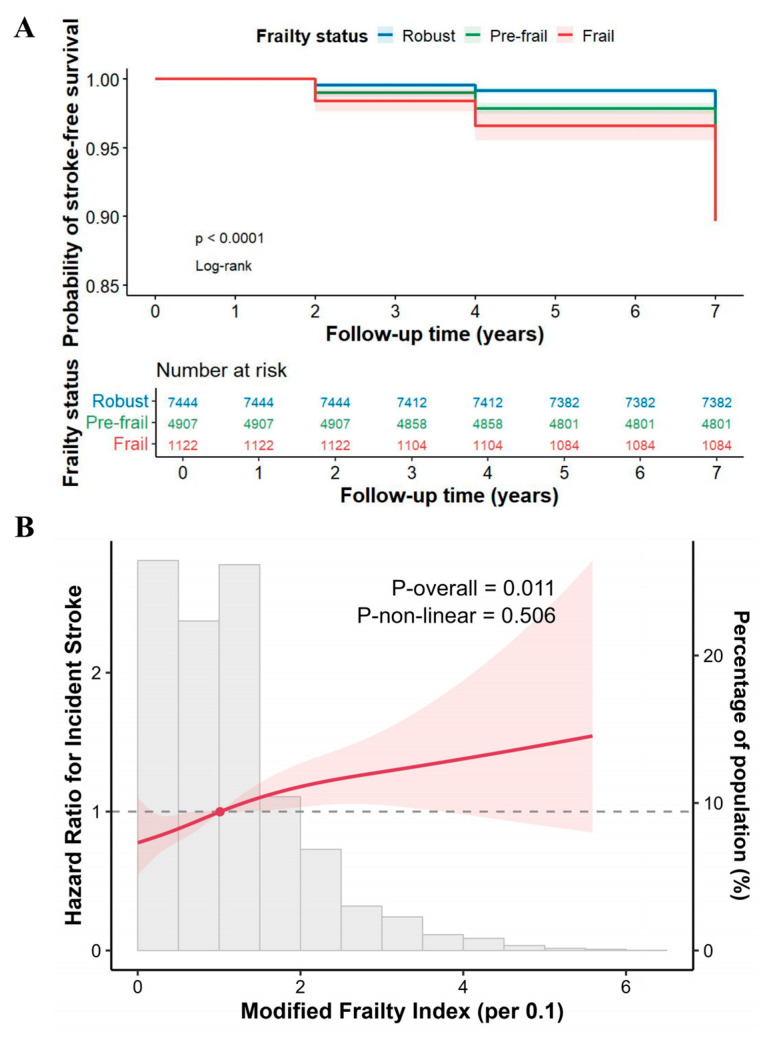
Association of frailty index (FI) with incident stroke in the CHARLS cohort. (**A**) Kaplan–Meier curves of stroke-free survival according to baseline FI categories. (**B**) Restricted cubic spline showing the association between the modified frailty index (mFI) and incident stroke risk; the red solid line represents the estimated hazard ratio, the shaded area represents the 95% confidence interval, the horizontal dashed line indicates the reference value, and the histogram shows the distribution of participants.

Kaplan–Meier analysis revealed significant differences in stroke-free survival among baseline frailty categories (log-rank *p* < 0.0001). Throughout follow-up period, participants classified as frail and pre-frail groups consistently demonstrated lower stroke-free survival rates compared to those in the robust group (Figure 2A).

Additionally, the restricted cubic spline analysis revealed a significant overall association between the mFI and stroke risk (*p* for overall association = 0.0112). There was no evidence of non-linearity (*p* for non-linearity = 0.5056), indicating an approximately linear positive relationship (Figure 2B).

### 3.2. Stroke Burden in China Based on GBD 2021

#### 3.2.1. Temporal Trends in Stroke Incidence in China

**Figure 3:**
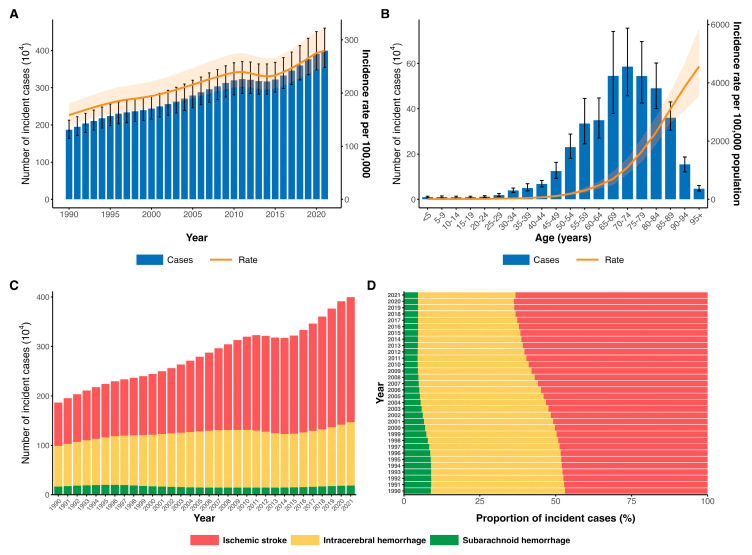
Temporal trends, age distribution, and subtype composition of incident stroke in China, 1990–2021 based on the GBD 2021 study. (**A**) Trends in incident cases and crude incidence rate, 1990–2021. (**B**) Age-specific incident cases and crude incidence rate in 2021. In panels (**A**,**B**), bars represent incident cases and lines represent crude incidence rates; error bars indicate 95% uncertainty intervals (UIs). (**C**) Trends in incident cases of ischemic stroke (red), intracerebral hemorrhage (yellow), and subarachnoid hemorrhage (green), 1990–2021. (**D**) Proportional distribution of stroke subtypes over time.

From 1990 to 2021, the overall number of incident stroke cases and the crude incidence rate in China exhibited a significant increase (Figure 3A; Table S4). The number of incident cases rose from 1.867 million to 3.996 million, more than doubling over the study period, while the crude incidence rate increased from 158 to 280 per 100,000 population. Despite minor fluctuations between 2012 and 2015, the overall trend indicates a sustained rise in the stroke burden over the past three decades.

#### 3.2.2. Age-Specific Distribution of Stroke Incidence

In 2021, stroke incidence exhibited a marked increase with advancing age (Figure 3B; Table S5). The burden remained low prior to age 45 but rose sharply thereafter. The number of incident cases increased from approximately 68,000 in the 40–44 age group to 125,000 in the 45–49 age group (≈1.8-fold), with the crude incidence rate rising from 74 to 114 per 100,000 population. With further aging, both metrics continued to rise: incident cases peaked at approximately 586,000 in the 70–74 age group, while the crude incidence rate reached its highest level among individuals aged ≥95 years (≈4561 per 100,000 population).

#### 3.2.3. Trends in Stroke Subtypes

From 1990 to 2021, the number of incident cases increased across all major stroke subtypes (Figure 3C; Table S6). Ischemic stroke remained the predominant subtype and exhibited the largest increase, rising from approximately 876,000 to 2.527 million cases. Intracerebral hemorrhage increased from approximately 825,000 to 1.285 million cases, whereas subarachnoid hemorrhage showed only a slightly increase (≈166,000 to 185,000 cases).

The proportions of stroke subtypes also shifted over time (Figure 3D; Table S7). The proportion of ischemic stroke increased from 46.9% to 63.2%, establishing it as the dominant subtype, while the proportion of intracerebral hemorrhage declined from 44.2% to 32.2%. Subarachnoid hemorrhage remained the least common subtype, decreasing from 8.9% to 4.6%.

### 3.3. Genetic Causal Inference: Mendelian Randomization Analysis

#### 3.3.1. Causal Effects of FI on Ischemic Stroke and Its Subtypes

The primary inverse variance weighted (IVW) analysis indicated that the genetically predicted frailty index (FI) was significantly associated with an increased risk of ischemic stroke. Specifically, each 1 standard deviation increase in FI corresponded to a 19.1% higher risk (OR = 1.191, 95% CI: 1.046–1.357; FDR < 0.05).

**Figure 4:**
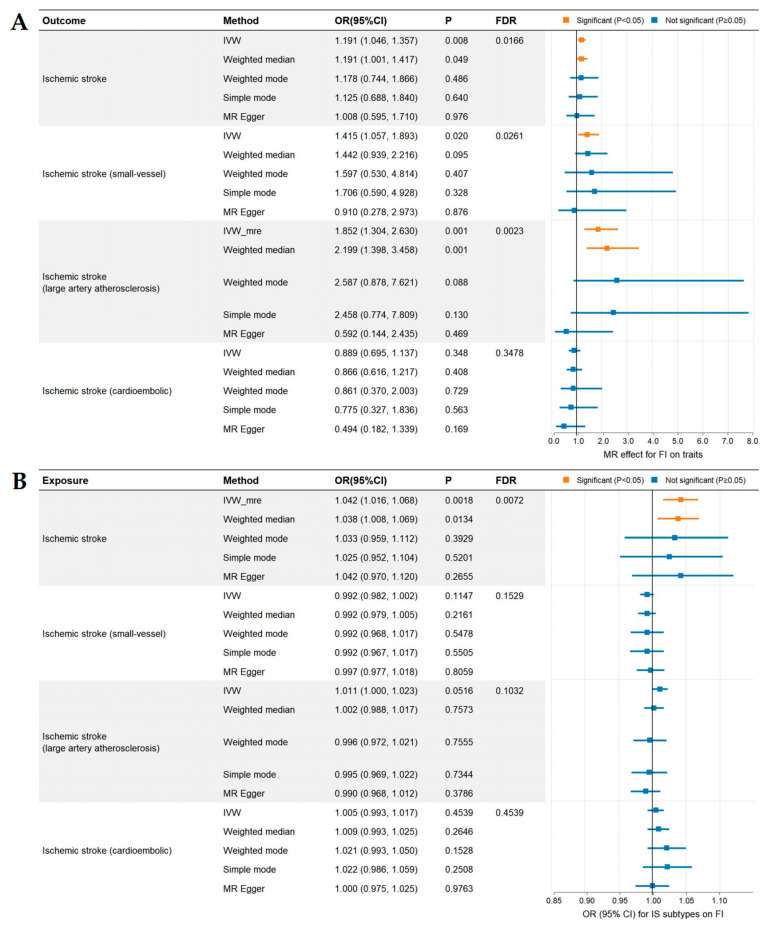
Bidirectional Mendelian randomization analyses of frailty index (FI) and ischemic stroke. (**A**) Causal effects of genetically predicted FI on ischemic stroke and its subtypes (LAS, SVS, and CE). (**B**) Reverse effects of genetic liability to ischemic stroke and its subtypes on FI. Estimates are presented as odds ratios (ORs) with 95% confidence intervals (CIs), using IVW as the primary method (IVW-MRE when heterogeneity was present). The vertical line indicates the null value (OR = 1.0). Abbreviations: CE, cardioembolic stroke; LAS, large-artery atherosclerotic stroke; SVS, small-vessel stroke; CI, confidence interval; FI, frailty index; IVW, inverse-variance weighted; IVW-MRE, multiplicative random-effects IVW; OR, odds ratio.

Subtype analyses revealed heterogeneity in these associations. Genetically predicted FI was positively associated with small-vessel stroke (OR = 1.415, 95% CI: 1.057–1.893; *p* = 0.0196). For large-artery atherosclerotic stroke, significant heterogeneity was detected (Cochran’s Q *p* < 0.05). The multiplicative random-effects IVW model was then applied, revealing a stronger association (OR = 1.852, 95% CI: 1.304–2.630; *p* = 0.0006). In contrast, no significant association was observed for cardioembolic stroke (OR = 0.889, 95% CI: 0.685–1.154; *p* = 0.374) (Figure 4A).

#### 3.3.2. Reverse MR and Multivariable MR Analyses

The reverse Mendelian randomization analysis suggested a modest effect of ischemic stroke on FI (IVW-MRE: OR = 1.042, 95% CI: 1.005–1.081; *p* = 0.027), although the magnitude was substantially smaller than that observed in the forward analysis (1.042 vs. 1.191). No significant reverse associations were detected for stroke subtypes (all *p* > 0.05). Overall, these findings support the notion that FI is the primary direction of the causal relationship (Figure 4B).

In the multivariable Mendelian randomization analysis, the direct effect of FI on ischemic stroke remained significant after adjusting for genetically predicted body mass index (BMI) and systolic blood pressure (SBP) (β = 0.290, SE = 0.105; *p* = 0.006).

#### 3.3.3. Sensitivity Analyses

Multiple sensitivity analyses reinforced the robustness of the findings. Cochran’s Q test was employed to evaluate heterogeneity among instrumental variables, while the MR-Egger intercept and MR-PRESSO analyses revealed no evidence of horizontal pleiotropy. The leave-one-out analysis demonstrated that the causal estimates were not influenced by any single SNP. Additionally, scatter plots and funnel plots further corroborated the stability of the results (Supplementary Table S8 and Figure S1).

## 4. Discussion

This study systematically evaluated the association between the frailty index (FI) and stroke across various levels by integrating a prospective cohort, disease burden analysis, and genetic causal inference. The findings from the prospective cohort indicated that an increased FI was positively and consistently associated with an elevated risk of stroke. This association remained significant after multivariate adjustments and multiple sensitivity analyses. Furthermore, a continuous dose–response relationship was observed between the FI and the risk of stroke. According to disease burden data, the incidence of stroke in China has steadily increased over the past three decades, particularly among individuals aged 45 years and older, with ischemic stroke progressively becoming the predominant type. Mendelian randomization analyses provided genetic evidence supporting a potential causal relationship between the FI and ischemic stroke.

The present findings elucidate the relationship between the FI and stroke risk. The FI, derived from the frailty accumulation model, serves as an indicator that reflects the cumulative impact of multiple health issues at the individual level. An increase in the FI often signifies a decline in the overall functional status of the body [35]. Such changes are not confined to a singular system; rather, they are likely to simultaneously influence the stability of vascular structures, metabolic regulatory capabilities [36], and levels of inflammatory response, thereby heightening the likelihood of cerebrovascular events across various levels. In contrast to isolated risk factors, the FI provides a more comprehensive depiction of an individual’s overall health status. Previous studies have indicated a correlation between frailty and adverse cardiovascular outcomes; however, evidence specifically linking frailty to stroke remains relatively limited, primarily derived from observational studies [37]. To address this gap, our study integrates prospective follow-up and genetic analysis, providing complementary evidence from observational and genetic perspectives. Notably, Mendelian randomization analysis mitigates the influence of confounding factors and reverse causality to a certain extent [38], supporting the possibility that the FI may contribute to stroke risk, rather than solely reflecting underlying disease burden.

In conjuction with the Global Burden of Disease (GBD) results from this study, the identified association holds practical significance within the current demographic context. As the incidence of stroke, particularly ischemic stroke, continues to escalate in China, the risk formation process may increasingly rely on long-term alterations in vascular structure and function [39]. In this regard, the global status indicated by the FI is likely more closely associated with stroke types that are rooted in vascular disease. This study demonstrates a more pronounced association with large-artery atherosclerotic stroke and small-vessel stroke, while no significant relationship was found with cardioembolic stroke [40].

On this basis, the significance of this study extends beyond merely adding a new association result; it offers a novel perspective on understanding risk. Previous assessments of stroke risk predominantly relied on individual risk factors, such as hypertension, diabetes, or dyslipidemia, which often reflect only specific abnormal states [41]. The findings of this study indicate that the FI encapsulates the overall condition resulting from the long-term accumulation of multiple functional changes, and the information it conveys cannot be entirely substituted by any single risk factor [5]. Furthermore, the integration of prospective cohort, disease burden, and genetic analyses strengthened the evidence supporting the observed association across complementary analytical frameworks. These findings may have important implications for the early identification and prevention of stroke.

The present findings may also have implications for stroke risk assessment and prevention strategies. Given that the FI reflects the cumulative burden of deficits across multiple physiological systems, its incorporation into existing stroke risk assessment frameworks may improve the identification of individuals who are vulnerable but may not yet meet conventional high-risk criteria based solely on individual vascular risk factors. From a public health perspective, routine frailty assessment could facilitate earlier recognition of high-risk populations and support the development of more personalized prevention strategies. Moreover, because the FI reflects a multidimensional health state that may be improved through interventions such as physical activity promotion, nutritional optimization, and comprehensive geriatric management, the FI may represent not only a marker of stroke risk but also a useful indicator for identifying individuals who could benefit from preventive interventions.

This study presents several limitations. First, stroke outcomes were primarily based on self-reported physician diagnoses, which may introduce a risk of misclassification. However, previous validation studies have demonstrated that self-reported stroke has acceptable validity in population-based epidemiological research, with reported positive predictive values of approximately 79% [42]. Nevertheless, some degree of outcome misclassification cannot be excluded. If present, such misclassification is likely to be non-differential and may have attenuated the observed associations. Second, the FI was constructed using existing survey variables, which may not comprehensively capture all health deficiencies. Third, the genetic data used in the Mendelian randomization analysis predominantly originated from populations of European ancestry. Although this may limit the direct generalizability of the findings to the Chinese population, the relationship between the frailty index and ischemic stroke is unlikely to be specific to a single ancestry group. Nevertheless, differences in genetic architecture across ancestry groups may influence the magnitude of the estimated effects. Variations in allele frequencies, linkage disequilibrium patterns, and gene–environment interactions may affect both the strength and validity of the genetic instruments, potentially leading to population-specific causal estimates. Furthermore, despite the various confounding adjustments and sensitivity analyses conducted in this study, the influence of residual confounding cannot be entirely ruled out.

Several important research questions arise from the present findings. First, the biological mechanisms linking the FI to stroke risk require further clarification, particularly regarding the roles of chronic inflammation, vascular aging, endothelial dysfunction, and metabolic dysregulation. Second, the stronger associations observed for ischemic stroke and specific ischemic stroke subtypes warrant further investigation to determine whether distinct pathophysiological mechanisms underlie these differences. Third, prospective intervention studies are needed to evaluate whether improvements in the FI achieved through lifestyle, nutritional, or multidisciplinary interventions can reduce stroke risk. Finally, large-scale genetic studies in East Asian populations are warranted to validate and extend the present Mendelian randomization findings in non-European ancestry groups.

## 5. Conclusions

The results of this study suggest that a higher frailty index (FI) is associated with an increased risk of incident stroke among Chinese adults. This association remains robust across various analytical methods. Furthermore, Mendelian randomization analyses provide genetic evidence supporting a potential causal association between the FI and ischemic stroke. Given the context of the rising burden of stroke and the increasing prevalence of ischemic stroke in China, the FI serves as a valuable indicator of overall health status. It may help identify individuals at higher risk of stroke and provide additional information for stroke prevention strategies.

## Figures and Tables

**Table 1:** Baseline characteristics by frailty index categories in the CHARLS cohort (N = 13,473).

	Total (*n* = 13,473)	Robust (*n* = 7444)	Pre-Frail (*n* = 4907)	Frail (*n* = 1122)	*p*
Age	59.49 (9.70)	56.87 (8.49)	61.81 (9.78)	66.75 (10.52)	<0.001
Gender					<0.001
Female	7099 (52.7)	3602 (48.4)	2792 (56.9)	705 (62.8)	
Male	6374 (47.3)	3842 (51.6)	2115 (43.1)	417 (37.2)	
Education					<0.001
divorced	183 (1.4)	100 (1.3)	65 (1.3)	18 (1.6)	
married	11,682 (86.7)	6754 (90.7)	4098 (83.5)	830 (74.0)	
unmarried	112 (0.8)	53 (0.7)	45 (0.9)	14 (1.2)	
widowed	1496 (11.1)	537 (7.2)	699 (14.2)	260 (23.2)	
Smoking					<0.001
Non-smoker	8127 (60.3)	4324 (58.1)	3086 (62.9)	717 (63.9)	
Ex-smoker	1155 (8.6)	534 (7.2)	481 (9.8)	140 (12.5)	
Smoker	4191 (31.1)	2586 (34.7)	1340 (27.3)	265 (23.6)	
Drinking					<0.001
None of these	9056 (67.2)	4629 (62.2)	3526 (71.9)	901 (80.3)	
Drink but less than once a month	1061 (7.9)	677 (9.1)	327 (6.7)	57 (5.1)	
Drink more than once a month	3356 (24.9)	2138 (28.7)	1054 (21.5)	164 (14.6)	
BMI					<0.001
Normal	6880 (53.0)	4320 (59.4)	2122 (45.2)	438 (42.7)	
Underweight	905 (7.0)	308 (4.2)	451 (9.6)	146 (14.2)	
Overweight	3731 (28.7)	2007 (27.6)	1425 (30.4)	299 (29.1)	
Obese	1472 (11.3)	636 (8.7)	693 (14.8)	143 (13.9)	
Hypertension					<0.001
no	8065 (59.9)	5243 (70.4)	2407 (49.1)	415 (37.0)	
yes	5408 (40.1)	2201 (29.6)	2500 (50.9)	707 (63.0)	
Diabetes mellitus					<0.001
no	12,467 (92.5)	7181 (96.5)	4380 (89.3)	906 (80.7)	
yes	1006 (7.5)	263 (3.5)	527 (10.7)	216 (19.3)	
Dyslipidemia					<0.001
no	10,274 (76.3)	6052 (81.3)	3481 (70.9)	741 (66.0)	
yes	3199 (23.7)	1392 (18.7)	1426 (29.1)	381 (34.0)	
Cardiovascular disease					<0.001
no	11,900 (88.3)	7201 (96.7)	3986 (81.2)	713 (63.5)	
yes	1573 (11.7)	243 (3.3)	921 (18.8)	409 (36.5)	
CES-D score	7.00 [3.00, 12.00]	6.00 [3.00, 10.00]	9.00 [5.00, 14.00]	14.00 [9.00, 20.00]	<0.001
Frailty index	0.09 [0.06, 0.16]	0.06 [0.03, 0.09]	0.15 [0.12, 0.19]	0.30 [0.27, 0.36]	<0.001

Abbreviations: BMI, body mass index; CES-D, Center for Epidemiologic Studies Depression Scale; *n*, number of participants.

**Table 2:** Associations of frailty index and modified frailty index with risk of incident stroke in the CHARLS cohort.

Model	HR (95% CI)	*p* Value
Model 1 (Unadjusted)	1.44 (1.35–1.54)	<0.001
Model 2 (Adjusted for age, sex)	1.41 (1.31–1.51)	<0.001
Model 3 (Fully adjusted)	1.16 (1.06–1.27)	0.0015
Sensitivity Analyses		
Exclude events within first 2 years	1.18 (1.07–1.3)	0.001
Exclude participants with baseline heart disease	1.22 (1.11–1.35)	<0.001

Model 1: crude model (per 0.1 increase in FI). Model 2: adjusted for age and sex (per 0.1 increase in FI). Model 3: fully adjusted model using mFI (per 0.1 increase), adjusted for educational level, marital status, smoking, alcohol consumption, BMI, depressive symptoms, hypertension, diabetes mellitus, dyslipidemia, and cardiovascular disease. Sensitivity analyses were based on Model 3.

## Data Availability

The datasets analyzed in this study are publicly available from the Global Burden of Disease (GBD) Results (https://vizhub.healthdata.org/gbd-results) (accessed on 29 June 2026).

## Supplementary Materials

The following supporting information can be downloaded at: https://www.mdpi.com/article/10.3390/healthcare14131932/s1, Figure S1. Sensitivity analyses for bidirectional Mendelian randomization between frailty index (FI) and ischemic stroke and its subtypes. Table S1. The 38 items used to construct the frailty index. Table S2. Summary of GWAS datasets used in this study. Table S3. Baseline Characteristics of included versus excluded participants in the CHARLS cohort. Table S4. Incident cases and crude incidence rates of stroke in China for all ages, 1990–2021 (GBD 2021). Table S5. Age-specific incident cases and crude incidence rates of stroke in China in 2021 by 5-year age groups. Table S6. Incident cases of ischemic stroke, intracerebral hemorrhage, and subarachnoid hemorrhage in China, 1990–2021 (all ages). Table S7. Proportional distribution (%) of stroke subtypes in China, 1990–2021 (all ages). Table S8. Sensitivity analysis of primary and secondary MR analyses.

## Abbreviations

CHARLS	China Health and Retirement Longitudinal Study
GBD	Global Burden of Disease
MR	Mendelian Randomization
IHME	Institute for Health Metrics and Evaluation
FI	frailty index
mFI	modified frailty index
IVW	inverse-variance weighted
IVW-MRE	multiplicative random-effects IVW
FDR	false discovery rate
CE	cardioembolic stroke
LAS	large-artery atherosclerotic stroke
SVS	small-vessel stroke
BMI	body mass index
SBP	systolic blood pressure
CI	confidence interval
OR	odds ratio
