# Supplementary material for: Frailty Index and Risk of Ischemic Stroke in China: Evidence from a Cohort Study, Disease Burden Analysis, and Mendelian Randomization
Source: Healthcare (Basel). 2026 Jul 1;14(13):1932. doi: 10.3390/healthcare14131932 (PMC13361964; doi:10.3390/healthcare14131932)
Supplement: Supplementary file 1 [file healthcare-14-01932-s001.zip › healthcare-4363648-supplementary.pdf]

Table S1. The 38 items used to construct the frailty index.

No	Description of the 38items	Cut-off value
1	Self-reported physician diagnosed hypertension	Yes = 1, No = 0
2	Self-reported physician-diagnosed dyslipidemia	Yes = 1, No = 0
3	Self-reported physician-diagnosed diabetes or high blood glucose	Yes = 1, No = 0
4	Self-reported physician-diagnosed cancer	Yes = 1, No = 0
5	Self-reported physician-diagnosed chronic lung disease	Yes = 1, No = 0
6	Self-reported physician-diagnosed liver disease	Yes = 1, No = 0
7	Self-reported physician-diagnosed heart disease	Yes = 1, No = 0
8	Self-reported physician-diagnosed kidney disease	Yes = 1, No = 0
9	Self-reported physician-diagnosed digestive disease	Yes = 1, No = 0
10	Self-reported physician-diagnosed psychiatric disorder	Yes = 1, No = 0
11	Self-reported physician-diagnosed memory-related disorder	Yes = 1, No = 0
12	Self-reported physician-diagnosed arthritis	Yes = 1, No = 0
13	Self-reported physician-diagnosed asthma	Yes = 1, No = 0
14	Self-reported chest pain (difficulty with climbing stairs, etc.)	Yes = 1, No = 0
15	Difficulty with physical disability	Yes = 1, No = 0
16	Difficulty with cognitive disability	Yes = 1, No = 0
17	Difficulty with vision impairment	Yes = 1, No = 0
18	Difficulty with hearing impairment	Yes = 1, No = 0
19	Difficulty with speech impairment	Yes = 1, No = 0
20	Difficulty with fine motor skills (e.g., picking up a coin)	Yes = 1, No = 0
21	Difficulty with dressing	Yes = 1, No = 0
22	Difficulty with bathing	Yes = 1, No = 0
23	Difficulty with doing housework	Yes = 1, No = 0
24	Difficulty with cooking	Yes = 1, No = 0
25	Difficulty with shopping	Yes = 1, No = 0
26	Difficulty with managing money	Yes = 1, No = 0
27	Self-reported pain	No pain = 0, Pain = 1
28	Difficulty with sleep	Yes = 1, No = 0
29	Activity limitation due to health (item 14)	Yes = 1, No = 0
30	Activity limitation due to health (item 13)	Yes = 1, No = 0
31	Activity limitation due to health (item 15)	Yes = 1, No = 0
32	Weak grip strength (below 20th percentile)	Yes = 1, No = 0
33	Slow gait speed (above 80th percentile for walking time)	Yes = 1, No = 0
34	Slow chair stand (above 80th percentile for chair rise time)	Yes = 1, No = 0
35	Difficulty with semi-tandem balance	Yes = 1, No = 0
36	Difficulty with tandem balance	Yes = 1, No = 0
37	Central obesity (waist circumference thresholds for men and women)	Yes = 1, No = 0
38	Underweight (BMI < 18.5)	Yes = 1, No = 0

Abbreviations: BMI, body mass index.

Table S2. Summary of GWAS datasets used in this study.

GWAS ID	Year	Traits	Samplesize	Ancestry	Consortium
ebi-a-GCST90020053	2021	Frailty index	175226	European	GWAS catalog
ebi-a-GCST006908	2018	Ischemic stroke	34217cases/ 406111controls	European	GWAS catalog
ebi-a-GCST006909	2018	Ischemic stroke (small-vessel)	5386cases/ 343560 controls	European	GWAS catalog
ebi-a-GCST006907	2018	Ischemic stroke (large artery atherosclerosis)	4373cases/ 297290 controls	European	GWAS catalog
ebi-a-GCST006910	2018	Ischemic stroke (cardioembolic)	7193cases/ 355468 controls	European	GWAS catalog
ieu-b-5138	2024	SBP	435859	European	UK Biobank
ukb-b-19953	2018	BMI	461460	European	MRC-IEU

Abbreviations: GWAS, genome-wide association study; SBP, systolic blood pressure; BMI, body mass index.

Table S3. Baseline characteristics of included versus excluded participants in the CHARLS cohort.

Variable	level	Excluded （N=4235）	Included （N=13473）	p
Age (mean (SD))		57.64 (11.39)	59.49 (9.70)	<0.001
Gender (%)	Female	2113 (50.3)	7099 (52.7)	<0.001
	Male	2086 (49.7)	6374 (47.3)	
Education (%)	Illiterate	975 (23.4)	3822 (28.4)	<0.001
	primary	1507 (36.2)	5437 (40.4)	
	middle school	925 (22.2)	2723 (20.2)	
	high school+	758 (18.2)	1485 (11.0)	
Marital Status (%)	divorced	46 (1.1)	183 (1.4)	<0.001
	married	3718 (88.3)	11682 (86.7)	
	unmarried	77 (1.8)	112 (0.8)	
	widowed	370 (8.8)	1496 (11.1)	
Smoking Status (%)	Non-smoker	2486 (59.0)	8127 (60.3)	<0.001
	Ex-smoker	262 (6.2)	1155 (8.6)	
	Smoker	1463 (34.7)	4191 (31.1)	
Drinking Status (%)	None of these	2715 (66.9)	9056 (67.2)	0.932
	Drink but less than once a month	319 (7.9)	1061 (7.9)	
	Drink more than once a month	1022 (25.2)	3356 (24.9)	
BMI Categories (%)	Normal	293 (47.1)	6880 (53.0)	0.007
	Underweight	40 (6.4)	905 (7.0)	
	Overweight	197 (31.7)	3731 (28.7)	
	Obese	92 (14.8)	1472 (11.3)	
Hypertension (%)	no	3017 (71.6)	8065 (59.9)	<0.001
	yes	1194 (28.4)	5408 (40.1)	
Diabetes Mellitus (%)	no	3914 (92.9)	12467 (92.5)	0.388
	yes	297 (7.1)	1006 (7.5)	
Dyslipidemia (%)	no	3366 (79.9)	10274 (76.3)	<0.001
	yes	845 (20.1)	3199 (23.7)	
Cardiovascular Disease (%)	no	3380 (80.3)	11900 (88.3)	<0.001
	yes	831 (19.7)	1573 (11.7)	
CES-D Score (median [IQR])		0.16 (0.13)	0.12 (0.09)	<0.001

Note: Data are presented as mean (SD) for continuous variables with normal distribution, median [IQR] for skewed variables (CES-D score), or n (%) for categorical variables.

Abbreviations: SD, standard deviation; IQR, interquartile range; BMI, body mass index; CES-D, Center for Epidemiologic Studies Depression Scale.

Table S4. Incident cases and crude incidence rates of stroke in China for all ages, 1990–2021 (GBD 2021).

Year	Number	Crude Rate
1990	1866788 (1642856 to 2125050)	158 (139 to 180)
1991	1953641 (1723085 to 2222288)	163 (144 to 186)
1992	2035307 (1796714 to 2314280)	168 (149 to 191)
1993	2109741 (1863301 to 2399994)	173 (153 to 197)
1995	2242854 (1976281 to 2557217)	182 (160 to 207)
1994	2179511 (1923573 to 2483216)	177 (157 to 202)
1998	2367248 (2089547 to 2698144)	189 (167 to 216)
1997	2336078 (2062332 to 2662941)	188 (166 to 214)
1996	2296147 (2025316 to 2618050)	185 (163 to 211)
1999	2401471 (2119229 to 2739192)	192 (169 to 218)
2001	2499568 (2205512 to 2854440)	198 (175 to 226)
2000	2443650 (2156115 to 2788293)	194 (171 to 222)
2002	2563465 (2262203 to 2929522)	202 (178 to 231)
2004	2711223 (2391226 to 3103267)	211 (186 to 242)
2003	2632800 (2323651 to 3011379)	206 (182 to 236)
2006	2875422 (2535181 to 3294192)	221 (195 to 253)
2005	2790804 (2459707 to 3196134)	216 (190 to 247)
2007	2961905 (2610483 to 3399569)	226 (199 to 259)
2009	3125943 (2754884 to 3585804)	235 (207 to 269)
2008	3043866 (2682152 to 3493205)	230 (203 to 264)
2010	3195475 (2814759 to 3666282)	239 (210 to 274)
2012	3212259 (2847492 to 3685962)	237 (210 to 272)
2011	3228118 (2851609 to 3704142)	240 (212 to 275)
2013	3178156 (2828557 to 3643356)	233 (207 to 267)
2015	3220164 (2880413 to 3675162)	233 (209 to 266)
2014	3170655 (2833200 to 3638008)	231 (206 to 265)
2016	3331645 (2975840 to 3806649)	239 (214 to 274)
2018	3604611 (3207949 to 4132910)	256 (227 to 293)
2017	3462207 (3087604 to 3962680)	247 (220 to 283)
2019	3765657 (3346038 to 4332414)	266 (236 to 305)
2020	3914822 (3476079 to 4506708)	275 (244 to 316)
2021	3996415 (3549619 to 4596679)	280 (249 to 322)

Table S5. Age-specific incident cases and crude incidence rates of stroke in China in 2021 by 5-year age groups.

Age	Number	Crude Rate
<5	9959 (7215 to 13964)	13 (9 to 18)
5-9	10318 (6311 to 15252)	11 (7 to 16)
10-14	9610 (5628 to 14318)	11 (7 to 17)
15-19	9716 (6303 to 14247)	13 (8 to 19)
20-24	11972 (8649 to 16236)	16 (12 to 22)
25-29	18673 (13497 to 25926)	22 (16 to 30)
30-34	38969 (30488 to 50157)	32 (25 to 41)
35-39	50114 (37521 to 69070)	47 (35 to 65)
40-44	68236 (55284 to 83750)	74 (60 to 91)
45-49	125349 (93135 to 163931)	114 (84 to 149)
50-54	230130 (181176 to 288153)	190 (150 to 238)
55-59	334843 (245332 to 445567)	305 (223 to 406)
60-64	349888 (270244 to 447601)	479 (370 to 613)
65-69	545466 (378920 to 741581)	707 (491 to 961)
70-74	586129 (457323 to 755620)	1098 (857 to 1416)
75-79	545050 (425795 to 697035)	1644 (1284 to 2102)
80-84	490028 (400837 to 601719)	2314 (1893 to 2842)
85-89	360555 (305463 to 430350)	3106 (2632 to 3707)
90-94	154236 (121634 to 186914)	3866 (3049 to 4685)
95+	47174 (36731 to 60750)	4561 (3551 to 5874)

Table S6. Incident cases of ischemic stroke, intracerebral hemorrhage, and subarachnoid hemorrhage in China, 1990–2021 (all ages).

Year	Ischemic stroke	Intracerebral hemorrhage	Subarachnoid hemorrhage
1990	876167 (720025 to 1075148)	825097 (674841 to 957755)	165524 (141571 to 191120)
1991	922183 (760109 to 1130362)	856622 (704929 to 985826)	174836 (150246 to 201654)
1992	966430 (799959 to 1183483)	886327 (733382 to 1021625)	182550 (157538 to 209649)
1993	1007512 (836953 to 1233232)	913468 (758466 to 1055895)	188761 (163315 to 216167)
1995	1045924 (869667 to 1280069)	939641 (781960 to 1084905)	193947 (168400 to 221576)
1994	1079895 (901114 to 1321857)	964748 (802232 to 1114167)	198211 (172407 to 226101)
1998	1110079 (926389 to 1356528)	987167 (822263 to 1140382)	198901 (172974 to 226670)
1997	1137548 (948071 to 1387248)	1004397 (837622 to 1160794)	194134 (168735 to 221508)
1996	1163617 (971012 to 1416369)	1017624 (849510 to 1176821)	186007 (161091 to 212742)
1999	1193506 (997130 to 1450665)	1030826 (861580 to 1192388)	177139 (153127 to 203093)
2001	1227671 (1026666 to 1489825)	1045869 (874451 to 1209604)	170109 (146932 to 195408)
2000	1271046 (1065650 to 1542381)	1063831 (889704 to 1231061)	164691 (142027 to 189217)
2002	1322311 (1107889 to 1604162)	1082213 (904543 to 1252776)	158941 (136907 to 182696)
2004	1378959 (1152776 to 1671103)	1100357 (919300 to 1273176)	153484 (131986 to 176169)
2003	1442484 (1208733 to 1740249)	1119603 (935028 to 1294761)	149136 (128274 to 170679)
2006	1506339 (1264875 to 1810943)	1137945 (949880 to 1316339)	146520 (125965 to 167236)
2005	1576536 (1327889 to 1891407)	1153532 (964050 to 1333462)	145355 (124915 to 165404)
2007	1653650 (1394132 to 1980526)	1163594 (973538 to 1342295)	144661 (124223 to 164807)
2009	1731726 (1457496 to 2070608)	1167814 (978434 to 1346695)	144326 (123644 to 164751)
2008	1811517 (1523032 to 2167041)	1169940 (980510 to 1351100)	144486 (123528 to 165013)
2010	1881659 (1580996 to 2253386)	1168884 (980285 to 1351083)	144932 (123693 to 165041)
2012	1926022 (1624253 to 2312042)	1156876 (971681 to 1334793)	145219 (124240 to 165243)
2011	1937124 (1629746 to 2328882)	1129942 (951029 to 1294150)	145193 (124545 to 164973)
2013	1933499 (1623505 to 2326657)	1099201 (928655 to 1257146)	145456 (125161 to 164937)
2015	1943538 (1631957 to 2337950)	1080284 (919492 to 1233552)	146833 (126607 to 166132)
2014	1985359 (1672826 to 2391689)	1085025 (928396 to 1239509)	149779 (129448 to 169251)

2016	2068542 (1735308 to 2498990)	1108393 (943784 to 1269636)	154709 (133264 to 174681)
2018	2169315 (1813833 to 2625691)	1132038 (959169 to 1299869)	160854 (137972 to 181657)
2017	2278884 (1899170 to 2768186)	1158156 (976660 to 1331924)	167570 (143138 to 189472)
2019	2396092 (1990542 to 2918916)	1194982 (1002717 to 1375945)	174583 (148617 to 197684)
2020	2497269 (2069630 to 3048373)	1237063 (1034969 to 1425613)	180489 (153421 to 205314)
2021	2526892 (2091834 to 3085131)	1284891 (1073803 to 1480724)	184632 (156940 to 210054)

Table S7. Proportional distribution (%) of stroke subtypes in China, 1990–2021 (all ages).

Year	Ischemic stroke(%)	Intracerebral hemorrhage(%)	Subarachnoid hemorrhage(%)
1990	46.93	44.2	8.87
1991	47.2	43.85	8.95
1992	47.48	43.55	8.97
1993	47.76	43.3	8.95
1995	47.99	43.11	8.9
1994	48.15	43.01	8.84
1998	48.35	42.99	8.66
1997	48.69	42.99	8.31
1996	49.15	42.99	7.86
1999	49.7	42.92	7.38
2001	50.24	42.8	6.96
2000	50.85	42.56	6.59
2002	51.58	42.22	6.2
2004	52.38	41.79	5.83
2003	53.2	41.3	5.5
2006	53.98	40.77	5.25
2005	54.83	40.12	5.06
2007	55.83	39.29	4.88
2009	56.89	38.37	4.74
2008	57.95	37.43	4.62
2010	58.89	36.58	4.54
2012	59.66	35.84	4.5
2011	60.3	35.18	4.52
2013	60.84	34.59	4.58
2015	61.3	34.07	4.63
2014	61.65	33.69	4.65
2016	62.09	33.27	4.64
2018	62.66	32.7	4.65
2017	63.22	32.13	4.65
2019	63.63	31.73	4.64
2020	63.79	31.6	4.61
2021	63.23	32.15	4.62

Table S8. Sensitivity analysis of primary and secondary MR analyses.

Exposure	Outcome	Number of IVs	Heterogeneity test		MR-Egger pleiotropy test		MR-PRESSO global pleiotropy test		
			Q	p-Value	Intercept	p-Value	RSSobs	p-Value	Outliers
Frailty index	Ischemic stroke	98	110.1893	0.1699	0.0035	0.5245	112.4808	0.1843	None
	Ischemic stroke (small-vessel)	98	104.3605	0.2866	0.0092	0.4528	106.5158	0.3033	None
	Ischemic stroke (large artery atherosclerosis)	98	133.7259	0.0080	0.0240	0.1063	124.483	0.0333	rs28453001、rs11245450、rs2477469
	Ischemic stroke (cardioembolic)	97	104.0432	0.2700	0.0123	0.2362	106.1614	0.2667	None
Ischemic stroke	Frailty index	43	75.2022	0.0012	-0.00004497	0.9865	79.0346	0.0013	None
Ischemic stroke (small-vessel)		29	21.2898	0.8131	-0.0013	0.5537	22.7260	0.8153	None
Ischemic stroke (large artery atherosclerosis)		28	39.0586	0.0626	0.0065	0.0423	42.2221	0.0673	None
Ischemic stroke (cardioembolic)		34	45.7179	0.0694	0.0009	0.4592	48.7918	0.0667	None

Abbreviations: MR, Mendelian randomization.

Figure S1. Sensitivity analyses for bidirectional Mendelian randomization between frailty index (FI) and ischemic stroke and its subtypes.

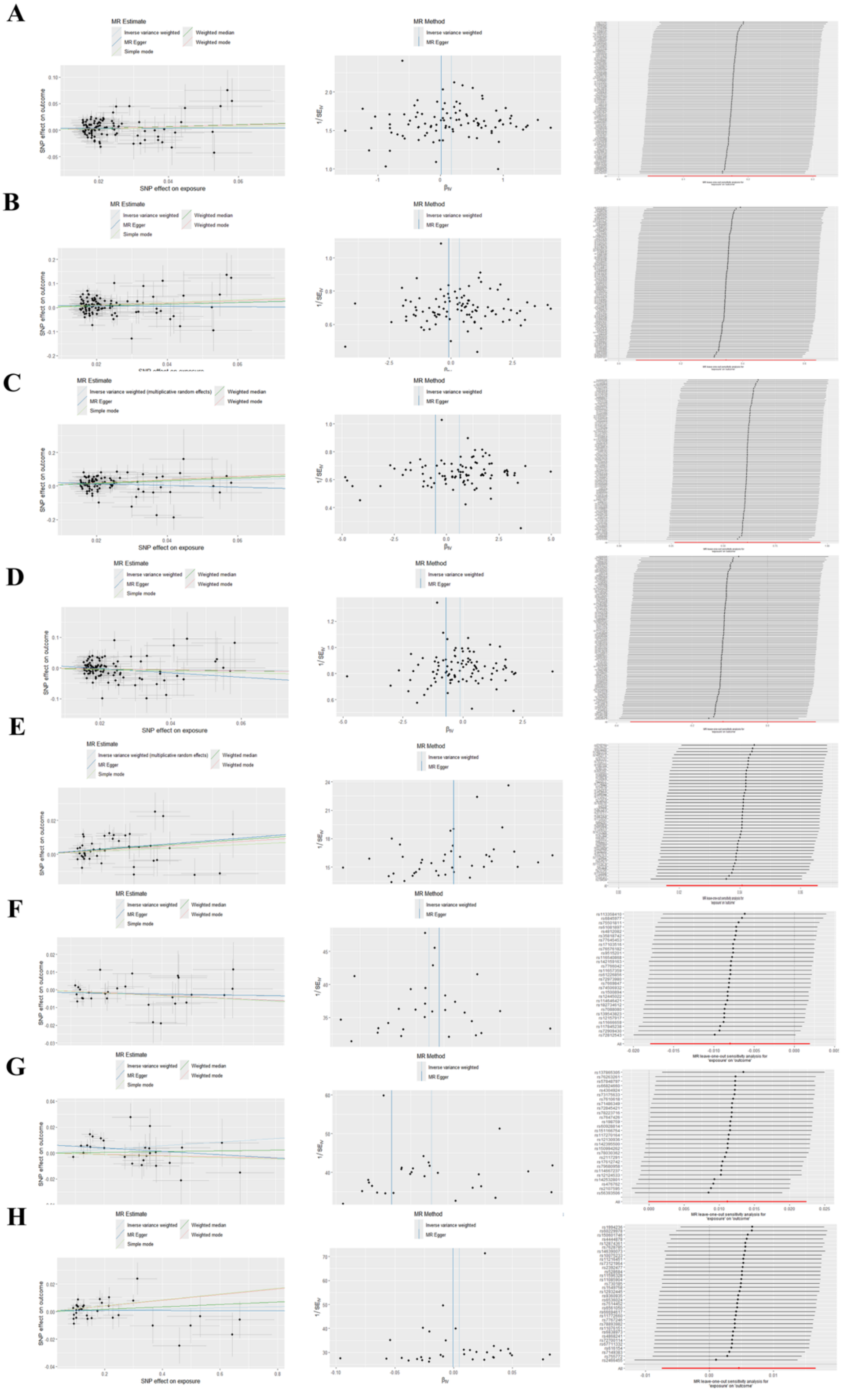

Note: Scatter plots, funnel plots, and leave-one-out analyses for bidirectional Mendelian randomization (MR). Panels (A–D) show the forward-direction MR analyses of genetically predicted frailty index (FI) on (A) ischemic stroke (IS), (B) small-vessel stroke (SVD), (C) large-artery atherosclerotic stroke (LAS), and (D) cardioembolic stroke (CE).

Panels (E–H) show the reverse-direction MR analyses of genetic liability to (E) IS, (F) SVD, (G) LAS, and (H) CE on FI. For each outcome, the scatter plot displays SNP-specific associations with the exposure and outcome, with regression lines corresponding to MR methods (as indicated). Funnel plots depict the Wald ratio estimates against their precision to assess potential directional pleiotropy. Leave-one-out forest plots show the IVW estimate after sequentially removing each SNP to evaluate the influence of individual instruments.
Abbreviations: CE, cardioembolic stroke; FI, frailty index; IVW, inverse-variance weighted; LAS, large-artery atherosclerotic stroke; MR, Mendelian randomization; SNP, single-nucleotide polymorphism; SVD, small-vessel stroke.
